# Nutrition Transition and Climate Risks in Nigeria: Moving Towards Food Systems Policy Coherence

**DOI:** 10.1007/s40572-020-00292-3

**Published:** 2020-10-01

**Authors:** Alexandra E. Morgan, Jessica Fanzo

**Affiliations:** 1 The Nitze School of Advanced International Studies, Johns Hopkins University, Washington, DC USA; 2 The Nitze School of Advanced International Studies, Bloomberg School of Public Health, Berman Institute of Bioethics, Johns Hopkins University, Washington, DC USA

**Keywords:** Nigeria, Food systems, Policy coherence, Nutrition transition, Climate change, Planetary health

## Abstract

**Purpose of Review:**

The purpose of this review is to describe the combined impacts of the nutrition transition and climate change in Nigeria and analyze the country’s national food-related policy options that could support human and planetary health outcomes.

**Recent Findings:**

This paper uses a food systems framework to analyze how the nutrition transition and climate change interact in Nigeria affecting both diets and the double burden of malnutrition, resulting in what has been termed the syndemic. Interactions between climate change and the nutrition transition in Nigeria are exacerbating diet-related inequities and will continue to do so if food systems continue on their current trajectory and without significant transformation. Siloed policy actions that attempt to mitigate one aspect of food system risk can create a negative feedback loop in another aspect of the food system. Our analysis finds that Nigeria has five national policies that include actionable steps to address food system insufficiencies; however, each of these policies is constrained by the boundaries of singular nutrition, climate change, and agricultural objectives. The country should consider a coherent policy environment that explicitly identifies and links underlying systemic and institutional drivers between climate change and malnutrition that simultaneously and comprehensively address both human and planetary health outcomes of food systems.

**Summary:**

The systemic and institutional outcomes of this emerging syndemic—undernutrition, obesity, and climate change—are inexorably linked. Nigeria lacks a coherent policy environment taking on this challenging syndemic landscape. The analysis in this paper highlights the need for Nigeria to prioritize their national nutrition and agricultural and climate policies that uncouple feedback loops within food systems to address climate change and malnutrition in all its forms.

## Introduction

The past two decades have produced a dynamic shift in world diets for low and lower middle-income countries [1•, 2]. Increased income and some significant interventions such as the promotion of exclusive breastfeeding, fortification of staple grains and salt, and sanitation and hygiene improvements are partially responsible for reductions in the worldwide burden of undernourishment from 14.8 to 10.8% [3]. However, urbanization, income shifts, and work and lifestyle patterns have simultaneously produced an increase in overweight and obesity across all regions [3].

As populations become more affluent, dietary consumption patterns shift. While diets become more diverse, moving away from meals composed predominantly with staple grains and/or tubers, there is also higher intake of refined carbohydrates and highly processed foods (and drinks) with higher amounts of added sugars, unhealthy fats, and sodium, and animal sourced foods. This typical dietary shift is known as the nutrition transition [4, 5]. This pattern is associated with globalization and urbanization factors but also an increasingly sedentary lifestyle [6], and the combination of these trends leads to shifts away from infectious diseases towards diet-related non-communicable diseases such as cardiovascular disease, diabetes, and stroke [4, 5].

What results from this transition is a population dealing with both undernutrition and overweight and obesity—what has been termed the double burden of malnutrition (also termed the multiple burdens of malnutrition) [1•]. The interaction between the nutrition transition and the epigenetic effects of undernutrition has fueled this trend. Early nutritional deficits put certain populations at risk for overweight and obesity later into adulthood [1, 7]. Both of these trends affect morbidity and mortality, and poor diets remain a high-risk factor for childhood death as well as adult non-communicable disease.

**Fig. 1:**
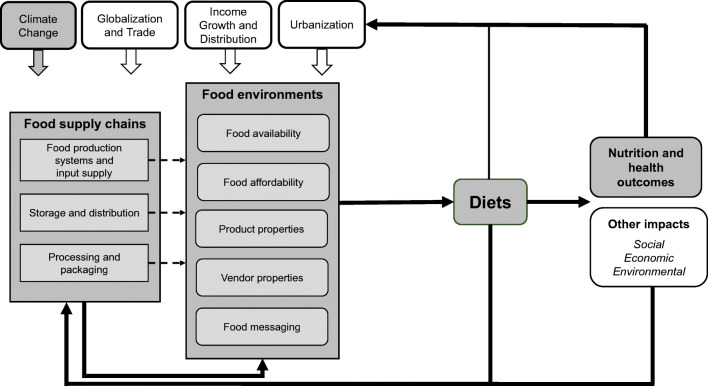
Food systems framework. Source: 35

Food systems connect and contribute to these dietary shifts, poor health outcomes, and environmental degradation [8•, 9••]. Unsustainable food production systems allocate calories and nutrients in inefficient ways, while also contributing to climate change [8•, 9••] and natural resource degradation [9••, 10••, 11]. Because of this circularity, climate change will continue to compound the health effects of malnutrition in all its forms [8•, 12–14]. The food systems framework (Fig. 1) highlights the interactions between environment, food, and health.

Climate change, undernutrition, and obesity share underlying societal causes, and feedback loops between them magnify negative health and nutrition outcomes. This “syndemic” is a synergy of epidemics that occur at the same time and place, interact, and share common drivers [15••]. These emerging syndemic risks exist in most countries but are particularly acute for the rural poor in the global South [16••, 17]. An increasing number of countries are experiencing a double burden of overweight and obesity alongside continued undernutrition, and unsustainable food systems are associated with both burdens [8•, 15••, 18]. If no action is taken to address climate change and its impacts on food systems, the historic progress made to reduce undernutrition could be lost, and overweight and obesity trends will continue to increase [1•, 8•, 15••]. Human and planetary health outcomes cannot be decoupled, and effective policies must target the feedback loops linking the health of both the planet and people [9••, 15••].

Nigeria is the most populous country in Africa, and its population of 196 million people [19] is expected to double by 2050 [20]. Within the Economic Community of West African States, Nigeria makes up more than two thirds of GDP [21]. Because of its economic growth and political status in the region, continent, and globally, guiding Nigeria towards a trajectory towards sustainable development is critical [21–23].

Nigeria is already experiencing the earlier described “syndemic.” Average maximum temperatures and average number of hot days have increased, while precipitation has decreased in the country [24]. Temperature change has led to increasing desertification in the north, erratic and changing rainfall across the country, and increased flood risk in coastal regions and along the major river systems [25–28]. These changes make food production, distribution, and utilization more vulnerable, which in turn affect dietary and nutrition outcomes [16••, 29]. At the same time, Nigeria is experiencing the double burden of malnutrition [30–32] with malnutrition ranked as the number one risk factor for death and disability, and sub-optimal diets have risen to become the 7th highest risk in the past decade [33]. Both climate change and the nutrition transition interact with the demographic shifts occurring in Nigeria, exacerbating dietary inequalities and affecting access to high quality, nutrient-dense, and safe food on a reliable basis.

This paper uses an adapted food systems framework (Fig. 1) from the High-Level Panel of Experts Report on Nutrition and Food Systems [34] to analyze the combined effects of climate change and the nutrition transition on Nigerian diets, examining multiple ways climate change will affect food supply chains and environments and its dietary and nutritional impacts on consumers. It also reviews the literature on two-way relationship of role climate change in accelerating the nutrition transition and vice versa in Nigeria and assesses to what degree current national food-related policies in Nigeria address the syndemic. Siloed actions that attempt to mitigate the effects of one issue can create potential negative feedback loops and impacts in another area [15••]. In order to address climate change and malnutrition, Nigeria must prioritize policies that address the syndemic—by uncoupling feedback loops between climate, undernutrition, and obesity by strengthening food system actions.

## Food Systems Framing

Food systems are composed of the people and activities that play a part in growing, transporting, supplying, and, ultimately, eating food. These processes also involve elements that often go unseen, such as food preferences and resource investments. Food systems influence diets by determining what kinds of foods are produced and available in markets. They also influence what foods people want to eat and are able to access. As shown in Fig. 1, the different parts of the food system include food supply chains, food environments, and consumer behavior (not shown in this framework for simplification purposes). Food supply chains consist of the activities and actors that take food from production, storage and distribution, process and packaging, retail, and waste [34, 35]. Food environments are the physical, economic, political and socio-cultural surroundings, opportunities, and conditions that create everyday prompts, shaping people’s dietary preferences and choices as well as nutritional status [34, 36]. They can be markets, restaurants, or cafeterias. These different parts shape food systems and can lead to both positive and negative outcomes across a range of outcomes [34].

Food systems are not static. They are in constant transition and are shaped and shifted by different internal and external drivers that push or pull the system in different directions [37]. While Fig. 1 does not show all the myriad drivers that shape food systems, climate change, urbanization, and globalization are significant drivers that instigate feedback loops of food system outcomes—diets, nutrition and health, environment, and livelihoods—on food supply chains and food environments. For the purposes of this paper, we are focusing on the gray-shaded elements and relationships of Fig. 1.

Climate change and natural resources shape food system functionality, efficiency, and resiliency [38]. In turn, food systems are a cause of climate change and environmental degradation [39]. Food systems contribute to greenhouse gas emissions, depletion of freshwater resources, deforestation, and nutrient pollution on landscapes and waterways [12]. The diversity, safety, and quality of diets very much depend on thriving food systems. However, evidence suggests that sub-optimal diets dominate across the world, contributing to both environmental and climate degradation and detrimental health outcomes [9••].

## Nutrition Transition of Nigeria

Malnutrition in all its forms is the number one risk factor for death and disease in Nigeria [33]. The combined effects of rising obesity and stagnating stunting in children indicate that Nigeria is going through a nutrition transition. Demographic and Health Survey (DHS) data have shown slight declines in stunting for children and adult female thinness as well as increasing adult female overweight and obesity over the last 20 years [40–44]. Independent studies have found that urban obesity increased by 20% in Nigeria between 2002 and 2010 [45]. While some populations have access to sufficient calories, stunting and thinness have decreased slowly, and undernourishment overall has risen since 2000, indicating that addressing malnutrition will require multiple strategies that span health, food, care, and the environment [46–48].

Both rural-urban and north-south divisions are characterized by different nutritional challenges in Nigeria. While the population is evenly split between rural and urban residents [49], currently, urban populations are more likely to experience obesity and overweight while rural populations are more likely to experience undernutrition [45]. However, evidence suggests that rural places are beginning to catch up with urban obesity trends [50]. At the same time, DHS data show that in the last 20 years underweight and vitamin deficiencies have been higher in the northern arid zones of the country, while overweight and obesity have been higher in the southern zones. Although northern zones produce more food for the country, residents in the north are the most likely to be food insecure [23]. Childhood stunting and undernutrition are particularly acute in northern Nigeria. Almost half of all children in the northeast and northwest are stunted [51].

Currently, Nigeria cannot meet food requirements through domestic production [52]. The major food crops produced in the country include cassava, cowpea, and sorghum [31, 53]. The Government of Nigeria (GON) reports that demand exceeds supply by more than 50% for rice, wheat, fish, milk, and tomatoes. Nigeria also underproduces maize, chicken, yams, and oil palm to a lesser extent [52] and has significant losses of fruits and vegetables as these perishable foods move along the supply chain [54]. Because it cannot meet its population’s demand with its own production, Nigeria imports between $3 and $5 billion worth of food annually [55].

Food imports both reduce Nigeria’s foreign exchange reserves and make consumers vulnerable to international prices. Over half of the population lives on less than $2 per day, including the majority of rural residents [4]. While some smallholder farmers produce enough food for family consumption, the average rural family only produces a quarter of food they consume [18, 56]. Across the country, the average household spends 65% of its income on food expenditure [57]. The unaffordability of nutritious food can be a significant barrier to consuming a healthy diet. The cost of a nutritionally adequate diet (CoNA) measures the minimum daily cost of meeting nutrient and energy requirements for a reference healthy adult woman, as a percent of daily per capita household spending on food and non-alcoholic beverages. In Nigeria, the CoNA is 92%, whereas in South Africa it is 42% and in the USA it is 32% [53].

While GON analysis focuses mainly on staple crops, low consumption of fruits and vegetables are also associated with negative health outcomes, and the combination of both domestically produced and imported fruits and vegetables in Nigeria do not meet the dietary requirements for daily vegetable consumption [58].

**Fig. 2:**
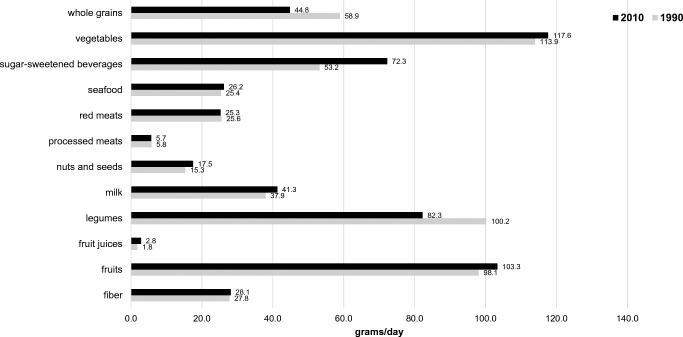
Changes in the Nigerian diets, 1990–2010. Source: [59]

Figure 2 shows how diets have changed in Nigeria comparing data from 1990 to 2010 (the most recent available) from the Global Dietary Database. There are interesting trends. Whole grains and legumes have decreased from 1990 to 2010, and sugar-sweetened beverages have increased. There are increases, although small, in the consumption of milk and fruits. This data source does not capture fish and seafood consumption, but these food sources are important components of the Nigerian diet. Overall, the health of the diet has not changed much over the last 15 years. Across the entire country, only 56% of women meet the minimum criteria for dietary diversity, a marker for dietary quality, in the previous day [40].

## Climate Vulnerability for Nigeria and Nigeria’s Food System

Temperatures in Nigeria are estimated to rise between 1.1 and 2.6 °C by 2060 [60] leading to losses of up to 30% of GDP [61]. Sea level rise is expected to reach 1 m by 2050 [27] affecting coastal regions and river systems. Temperature increases will affect agriculture mainly through changes in rainfall. Climate change is projected to make the northern zones drier and the southern zones more wet [27, 28, 60] Increasing desertification will harm the northern part of the country, while sea level rise, saltwater intrusion, and flooding will affect the southern part of the country [62–64].

Climate change will impact Nigeria’s agro-ecological zones in different ways. Nigeria has two main types of vegetation—tropical rainforest in the south and savannah in the north. Erratic rainfall and unpredictable severe weather events may affect agricultural production across both areas. While specific climate risks will differ depending on the agro-ecological zone, most key challenges will be related to water resources. In the north, dryness and drought will affect both agricultural production and livestock, while in the south, soil porosity issues will heighten the effects of sudden increased rain and flooding [26]. Climate change will impact each zone differently but will also shift the boundaries between the zones [27], challenging traditional agricultural cultivation patterns. The Nigerian government predicts that the major impacts for agricultural production will include lower yields and poor livestock performance in the Sahel and savanna agro-ecological zones, rapid farm wastage, decreased soil fertility, and leaching. Seawater incursion and desertification will reduce arable land [29]. Flooding will also affect coastal and freshwater fisheries production negatively through siltation and contamination [61]. Some climate models suggest that rainfed agriculture may decline up to 50% by 2080, while overall agricultural productivity could decline by 10–25% [65].

Increasing temperature [63], shifts in rainfall, [64, 66] and atmospheric carbon dioxide concentration [60] will drive shifts in agricultural yield. Across West Africa, a hotter dry season will cause rice yields to decline, even with increased irrigation [67]. In Nigeria, an increase in the number of days with extreme temperature will reduce the output of cassava, cocoyam, sweet potatoes, cowpeas, and maize [63] By 2050, hotter temperatures are expected to reduce output of rice, sorghum, millet, maize, yam, and cassava [68]. Increased rainfall will also compromise sweet potato and rice production [64]. In the short term, warmer temperatures are associated with increased production of millet, onions, tomatoes, and melon in northern Nigeria [63]; however, erratic rainfall patterns may reduce any positive effects of temperature.

Nutrient content will also shift in major food crops and consumers’ access to nutrients will be negatively affected by changes to food storage. Increased concentrations of atmospheric carbon dioxide will reduce zinc, iron, and protein in rice [69] and cassava will have increased concentrations of cyanide [60]. Heat will also make storage of vegetables more difficult [29]. Consumers will also have elevated exposure to mycotoxins, such as aflatoxin, and mold increases because only 10% of maize traders across the country fumigate and dry their wares [64].

Increased surface water temperature, sea level rise, and ocean acidification will negatively affect aquaculture and fisheries [70, 71]. This will affect nutrient access for much of Nigeria since fish make up about ½ of protein intake [70]. Human control may make aquaculture more resilient to climate change [70]; however, farmed fish may hold less nutritional value than wild caught fish [72]. Nigeria is one of the several countries identified by Golden et al. as most reliant on fish, which also has estimated marine catch reductions of over 20% by 2050 [72].

Rainfall change will have particularly negative effects on agriculture because of Nigeria’s dependence on rainfed irrigation. Only 1% of agricultural land currently uses modern irrigation [26, 28, 73]. The north central region may have lower sensitivity to rainfall changes in the short run, if lake water in the region is harnessed to provide irrigation [26]. However, irrigation is not a panacea given uncertain changes in rainfall. Extreme weather may also cause flooding and a widespread lack of flood control will make erratic rain particularly damaging [29, 62].

The agricultural impact of severe flooding caused by heavy rains in 2012 provides an example of the multiple pathways in which climate change may affect household nutrition. In 2012, heavy rainfall strained dam infrastructure and flooded settlements along the Niger, Benue, and Gongola rivers, causing $16.9 billion dollars worth of damage [62]. The disaster destroyed 30% of the year’s rice production. The majority of families affected by floods that relied on agricultural production to meet their family’s nutritional needs were forced to turn to markets to buy the majority of their food [62]. Increased demand created a subsequent negative feedback loop elevating the price index for food and making market consumption more inaccessible. Scarcity coping strategies used by families included borrowing food, reducing or skipping meals, and relying on less preferred foods [62]. Countrywide surveys show that even absent crisis level shocks, many families resort to food coping strategies. In 2010 and 2011, countrywide surveys showed that between 7 and 9% of those in the lowest income quintile used severe coping strategies such as going to bed hungry or borrowing food from the community or local NGOs as food assistance in the past 7 days [56].

**Fig. 3:**
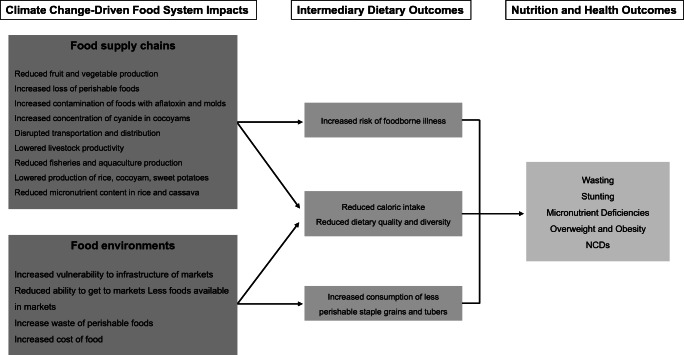
Impacts of climate change on food systems for Nigeria. Sources: [26, 27, 29, 60, 61, 63, 64, 67]

Pricing feedback loops highlight the economic effects of climate change. Lowered or shifted yields will impact livelihoods for 60% of Nigeria’s labor force involved in agricultural production, 90% of which are smallholder farmers [27]. The price effects of lowered yields will also affect non-farming and urban populations reducing their access to high quality and nutritious diets. Additional challenges affecting the food system include certain push factors such as increasing movement from rural to urban areas, putting more stress on food supply chains to urban areas and encroachment of urbanization into adjacent rural land (hinterlands) that are near major urban centers [26]. Figure 3 summarizes some effects of climate change on food system elements (focusing on food supply chains and food environments), intermediary diet outcomes, and nutrition and health outcomes in Nigeria using the food systems framework in Fig. 1.

## Assessment of Relevant Nigerian Food- and Climate-Related Policies

Using the food systems framework as a guide, we assessed five national policies in Nigeria, the Agriculture Promotion Policy (APP), the National Adaptation Strategy (NASPA-CCN), the National Policy on Food and Nutrition (NPFN), the Agriculture Sector Food Security and Nutrition Policy (ASFS), and the National Strategic Plan of Action on Nutrition (NSPAN), to determine whether they used a food systems framing to develop interventions targeting the syndemic—malnutrition, nutrition-sensitive agriculture, and climate change. Nigeria committed to the creation of a health and environment strategic alliance in the Libreville declaration, acknowledging the important link between environmental risks and health outcomes [74]. Overall, Nigeria has developed strong policy guiding documents and national strategies on climate change, nutrition, and agriculture. However, policy implementation has been limited due to budgetary constraints and stakeholder fragmentation [23, 75] with state budgets and policies not matching federal planning [23, 76].

The Nigerian government has made inroads to combine agriculture and nutrition policies using a systems perspective. However, the explicit integration of climate change adaptation and mitigation actions into nutrition policy has been limited. Three of 5 policies recognize the double burden of undernutrition and overweight/obesity, while two policies focus on undernutrition exclusively. The three nutrition polices have a weak focus on climate change, while the climate change policy has little focus on nutrition. All five policies make target interventions at various points along the supply chain, but agriculture production focused interventions are the most prevalent in the climate change and agriculture policies, while demand focused interventions to improve diets are stronger in the nutrition policies. Food environment interventions overall were limited with very little focus on how to change the affordability of diets or the choice architecture of these built places to facilitate healthier eating. More significantly, only 3 of the policies contain a cost analysis of implementing the goals and actions set out in those policies. All of the policies contained a monitoring and evaluation (M&E) plan. The most comprehensive set of interventions along the supply chain comes from the Agriculture Sector Food Security and Nutrition Policy; however, this plan does not explicitly integrate the effects of climate change on the supply chain. Table 1 below summarizes the results of the analysis.

**Table 1:** National policy environment in Nigeria

National strategy document	Years active	Does the policy address multiple burdens of malnutrition?	Does the policy address the ag-environment-nutrition nexus?	Does the policy include interventions along food systems?	Cost analysis	M&E plan
National Adaptation Strategy and Plan of Action (NASPA-CCN)	2011–current	No Undernutrition only	No •Focus on climate impacts on agricultural production •Focus on food access •No mention of nutritional quality of food	No Food supply chains •Improved agriculture systems for crops and livestock •Increase irrigation Food environment/markets •Facilitate credit to smallholder farmers	No	Yes* *Focused on building out framework, not interventions
Agriculture Promotion Policy (APP)	2016–2020	Yes Recognizes double burden of underweight, overweight, and obesity	No •Focus on agribusiness supply chains •Integrating climate-smart agriculture* •Integrating nutrition-sensitive programming* *Mentioned as a goal to integrate throughout all objectives, but many objectives lack integration of nutrition and climate	Yes Food supply chains •Fertilizer, increased soil nutrients •Irrigation •Pest and disease control services •National grain storage centers Food environment/markets •Infrastructure improvement Diets •Import substitution to reduce effects of exchange rate policy on consumers •School feeding programs •Strategic reserves of food	No	Yes
National Policy on Food and Nutrition (NPFN)	2016–Current	Yes Recognizes burden of malnutrition in all its forms	No •Links agriculture and food security •Does not mention climate change	Yes Food supply chains •Promote priority value chain crops •Improved on-farm storage •Myco-toxin prevention •Improve processing and preservation Food environments/markets •Effective food distribution systems •Improved infrastructure Diets •Biofortification of staple foods, vitamin provision •Food quality standards •National buffer stock to reduce price volatility •School feeding programs	No	Yes
Agriculture Sector Food Security and Nutrition Policy (ASFS)	2016–2025	Yes Recognizes burden of malnutrition in all its forms	No •Strong focus on agriculture and nutrition •Climate change mentioned once	Yes Food supply chains •Expand production of bio-fortified foods •Scale up fruit and vegetable production •Reduce post-harvest loss through increased cold storage and processing •National aflatoxin control initiative Food environments/markets •Facilitate access to credit for smallholder farmers •Improved nutrition labeling Diets •Develop improved food-based dietary guidelines	Yes	Yes
Health Sector Component of National Food and Nutrition Policy (NSPAN)	2014–2019	No Focus on undernutrition, specifically for mothers and children Does not address overweight and obesity	No •No mention of agriculture or climate change	No Diets •Vitamin supplementation •Feeding centers for severe acute malnutrition •Breastfeeding promotion	Yes	Yes

Sources: [30, 31, 52, 61, 77]

### Challenges to Implementation

While Nigeria’s policy documents highlight actionable items to create nutrition-sensitive and climate-smart interventions, significant challenges remain. The NASPA-CCN was developed in 2011, but fragmented implementation of the strategy has made farm-level adaptation to climate change difficult to achieve [78]. The agricultural section of the National Climate Policy focuses on the diversification of livestock, increased access to drought resistant crops, better soil management practices, national early warning systems, and increased use of irrigation and crop cover [61]. The government also suggests the increased provision of crop insurance at subsidized rates for smallholder farmers, provided by non-profits, or through public-private partnerships. However, adaptation results have been limited. While the climate policy has ostensibly been in effect for 9 years, a 2019 study in two northern communities found that while farmers were using adaptation strategies, they were not utilizing the strategies best suited for their dryland environment [79]. Irrigation policies are also critical for climate adaptation, but efforts to scale up irrigation have been fragmented and duplicative [73, 80].

Numerous surveys of Nigerian farmers have highlighted the continued challenges to increase the use of adaptation strategies. Studies of farmers’ perceptions of climate change have shown that farmers accurately perceive changes in temperature and rainfall [81], but climate adaptation strategies depend on farmer income, perception of risk, and environment [82–84]. Other surveys have found that adaptation strategies for farmers differ between older and younger farmers [82] and that in some communities, challenges remain regarding farmers’ perception of the causes of climate change. Farmers with larger farms, higher incomes, and more schooling are more likely to practice adaptation strategies [82] while those that link climate change to religious beliefs are less likely to proactively use adaptive strategies [81]. A significant information gap exists between farmers [83]. Without increasing expenditure on agriculture and other related industries such as aquaculture and agroforestry, smallholder farmers are likely to be left out of mitigation and adaptation strategies.

Nutrition policies also lack budget allocations. The Scaling up Nutrition (SUN) Movement reports that Nigeria has met 70% of the criteria for an enabling policy environment for nutrition as of 2019 [85]. However, budget allocations have not matched the goals outlined in the costed strategies. Surveys of government and NGO stakeholders in 2015 and 2016 found concerns about appropriate resource allocation at the federal and state level [86]. In 2019, Nigeria only spent 0.2% of its budget on nutrition specific interventions [85]. Budget delays lead to limited implementation. A review of federal stakeholders with responsibility for the NSPAN (2014–2019) found that only 1/3 had begun implementation of the plan by 2018 [87]. Limited domestic resource mobilization means that nutrition programs rely heavily on international donors for funding [85] and those resources can vary widely depending on donor motivations.

Strong policy guidance with a lack of implementation funding has been a continued problem for the agriculture, climate, and nutrition sectors in Nigeria. Increased government spending on agriculture has been linked to economic growth [88]. However, Nigerian budgets are heavily dependent on world oil prices [23, 26], and current price slumps jeopardize programming for agriculture and nutrition (Table 1).

## Conclusion and Recommendations

While budgetary dependence on oil revenues and the fragmentation of the federal system will challenge Nigeria’s ability to implement policies that focus on the common systemic drivers of malnutrition and climate change, Nigeria has made large strides to improve its policy environment and coherence. Its 2017 agricultural and nutrition policy highlights a systemic understanding of the drivers of malnutrition. Integrating climate change into a more coherent food systems framework would provide nuance to future assessments of malnutrition and will allow Nigeria to address and mitigate climate risks for nutrition. Doing so would require some major revisions to the policy. Ecker et al. (2020) also recommended that Nigeria’s policymakers consider adopting a food systems framework to reformulate national food and nutrition policies in order to improve household diets and reduce the multiple burdens of malnutrition [89].

One revision would be to fulfill the Comprehensive Africa Agriculture Development Program (CAADP) pledge to invest in agriculture, and, within agriculture, nutrition-climate-sensitive agriculture. Nigeria is a signatory to CAADP, which has set a goal of 10% of government budget going towards agriculture. However, Nigeria has consistently underperformed on this metric. Average yearly agricultural spending between 2008 and 2012 only totaled 4.6% of the national budget. [90]. The most recent CAADP performance assessment gave Nigeria 0% progress on its three goals related to modernized agriculture, well-nourished citizens, and environmentally sustainable and climate resilient economies [91] Nigeria must increase domestic funding for climate, agriculture, and nutrition in order to address malnutrition.

A second revision would be to consider double- or triple-duty actions. Double- and triple-duty actions simultaneously act on two or three of the epidemics (undernutrition, overweight, and/or climate). One example could be reducing red meat consumption to prevent cancer/obesity (obesity/NCDs), increase land efficiency to grow food for human consumption (undernutrition), and lower greenhouse gas emissions (climate change). These actions are carefully calibrated so that a focus on undernutrition does not lead to an increase in overweight and obesity for certain populations [92••]. Swinburn and colleagues articulated areas in which double- or even triple-duty actions could mitigate the sydemic, many with direct actions with food environments, the place where consumers engage with food systems [15••]. However, if Nigeria’s national policies that touch on food systems do not address the impacts of climate change, today’s investments may have little impact in the next three decades. An integrated, holistic food systems framing linking climate-smart and nutrition-sensitive agriculture on both land and water could identify priority areas for triple-duty actions to make a more significant impact on the syndemic in Nigeria [9••]. Some examples include scaling up nutrition-sensitive agriculture programs with climate adapted seeds and irrigation technologies, adapting food-based dietary guidelines to promote nutrient-dense and climate-conscious diets, taxing highly processed foods and devoting revenue streams to climate-smart agricultural policies, and developing more urban agriculture. While some of these elements are already present in Nigeria’s national policies, budgetary guidelines do not explicitly promote double- or triple-duty actions. Given Nigeria’s budget constraints and oil revenue volatility, double- or triple-duty actions should become priority strategies.
